# Association of circulating CEACAM1 levels and insulin sensitivity in gestational diabetes mellitus

**DOI:** 10.1186/s12902-020-00550-3

**Published:** 2020-05-15

**Authors:** Yiming Wu, Zhen Yang, Lingfei Zhu, Qing Su, Li Qin

**Affiliations:** 1 Department of Endocrinology, Xinhua Hospital Chongming Branch, Shanghai Jiao Tong University School of Medicine, 1665 Kongjiang Road, Shanghai, China; 2 Department of Endocrinology, Xinhua Hospital, Shanghai Jiaotong University School of Medicine, Shanghai, China

**Keywords:** Carcinoembryonicantigen-related cell adhesion molecule 1, Gestational diabetes mellitus, Insulin sensitivity

## Abstract

**Background:**

The aim of this study was to estimate the levels of circulating carcinoembryonic antigen-related cell adhesion molecule 1 (CEACAM1) in subjects with gestational diabetes mellitus (GDM) and investigate the relationships between CEACAM1 and GDM.

**Methods:**

Circulating CEACAM1 levels were measured by ELISA kit in 70 women with GDM and 70 normal glucose tolerance (NGT) pregnant women. Blood samples were collected to detect fasting plasma glucose (FPG), fasting insulin (FINS) and glycosylated hemoglobin (HbA1c) levels in all participants. Insulin sensitivity index (ISOGTT) was calculated to assess insulin sensitivity. Correlation analysis was performed between serum CEACAM1 levels and other parameters.

**Results:**

Circulating CEACAM1 levels were higher in the GDM group than that in the NGT pregnant group, however, the difference showed no statistical significance (1889.82 ± 616.14 vs 1758.92 ± 433.15 pg/ml, *p* > 0.05). In GDM group, CEACAM1 was positively correlated with ISOGTT (*R* = 0.39, *P* = 0.001), while negatively with 1 h post-meal plasma insulin level (1hPINS) (*R* = -0.32, *P* = 0.008), 2 h post-meal plasma insulin level (2hPINS) (*R* = -0.33, *P* = 0.006) and area under curve of insulin (AUCI) (*R* = -0.36, *P* = 0.002) when adjusting for maternal age and gestational age.

**Conclusions:**

The present study showed that circulating CEACAM1 levels did not differ in both GDM and NGT groups. However, we found a significant positively correlation between CEACAM1 and insulin sensitivity in the GDM group.

## Background

Gestational diabetes mellitus (GDM) is diabetes that is first diagnosed in the second or third trimester of pregnancy that is not clearly either preexisting type 1 or type 2 diabetes (T2DM) [1]. In recent decades, the prevalence of GDM has been increasing worldwide, especially in some developing countries [2]. Due to different diagnostic tests, diagnostic criteria, racial difference and so on, the prevalence of GDM reported in various countries ranged from 1.7 to 37.7% [3, 4]. GDM is a metabolic disorder during pregnancy which results in various adverse acute and chronic complications. Besides, both GDM women and the newborns may have an increased incidence risk of T2DM, obesity as well as metabolic syndrome in the future [5–7]. So far, the fundamental pathogenesis of GDM have not been clearly understood. However, IR is a well-documented hallmark of T2DM and GDM.

Recently, Carcinoembryonic antigen-related cell adhesion molecule one (CEACAM1) has been suggested as a potential regulatory molecules in the pathology of IR. CEACAM1 also known as CD66a, BGP, belongs to the carcinoembryonic antigen (CEA) family [8]. It expresses highly in liver, but not in classical insulin target peripheral tissues such as white adipose tissue and skeletal muscle [9]. Secreted insulin is mostly cleared in liver. Impairment of insulin clearance can be the primary cause of IR [10]. CEACAM1 is a substrate of the insulin receptor in liver, and it is phosphorylated in response to insulin in hepatocytes and then induces insulin clearance by increasing receptor-mediated endocytosis and degradation [11–13]. Regarding this, we hypothesize that CEACAM1 may play a protective role in the occurrence and development of GDM.

To our knowledge, there were no studies on the associations between CEACAM1 and GDM had been reported in the literature. In the present study, we aimed to investigate the circulating CEACAM1 levels and its possible relationship with GDM.

## Methods

### Subjects

From May to December 2015, pregnant women routinely tested for GDM with a 75-g oral glucose tolerance test (OGTT) between the 24th and 28th weeks of gestation in Xinhua Hospital Chongming Branch Affiliated to Shanghai Jiao Tong University School of Medicine. Pregnant women with multiple pregnancies, preexisting diabetes, hypertension, thyroid dysfunction or other endocrine diseases, acute or chronic inflammation were excluded. 70 pregnant women were newly diagnosed with GDM (GDM group, *n* = 70) according to the American Diabetes Association criteria. Another 70 subjects were randomly selected from normal glucose tolerance pregnant women (NGT group, *n* = 70) in accordance with the random number table. GDM was diagnosed according to the criteria of ADA if one or more plasma glucose levels were elevated during the OGTT with the following threshold glucose levels: fasting≥5.1 mmol/l; 1 h ≥ 10.0 mmol/l; 2 h ≥ 8.5 mmol/l. This study was approved by the Ethics Committee of the hospital and conformed to the provisions of the Declaration of Helsinki. All participants signed the informed consent.

### Clinical and biochemical assessment

We inquired and recorded the information of name, age, pre-pregnancy weight, gravidity and parity times, and family history of diabetes from all participants at the first prenatal examination between the 13th and 15th weeks of gestation. Anthropometric measurements including height, bodyweight, abdominal perimeter, and blood pressure were measured in the second trimester. Body mass index (BMI) was calculated as the weight divided by the squared height (kg/m^2^). Venous blood samples were collected after overnight fasting. The serum specimens were separated and stored at − 80°Cuntil analyses were performed. Venous plasma glucose level was measured by glucose oxidase method (ADVIA-1650 Chemistry System, Bayer, Leverkusen, Germany), and glycosylated hemoglobin was measured by high-performance liquid chromatography (BIO-RAD, Laboratories, CA). Serum insulin was measured by chemiluminescent immunoassay (ARCHITECT ci16200 analyzer, Abbott Laboratories, Illinois, USA). Circulating CEACAM1 levels were determined by ELISA kit (R&D Systems, Minneapolis, MN). The sensitivity of the assay was 93 pg/ml with inter- and intra-assay coefficients of variation of < 10 and < 9%, respectively. Homeostasis model assessment of insulin resistance (HOMA-IR) was calculated as follows: FPG (mmol/l) × FINS (mU/L)/22.5; insulin sensitivity index (ISOGTT) was calculated as [10,000/sqrt (FPG × FINS × mean glucose ×mean insulin]; HOMA-β reflecting the pancreatic β-cells function was calculated as [FINS× 20/ (FPG-3.5)].

### Statistical analysis

All analyses were performed using the Social Sciences software version 22.0 (SPSS, Chicago, IL). Measurement data were expressed as mean ± standard deviation (SD) or medians (inter-quartile ranges), comparisons of measurement data among groups were carried out by independent sample t-test method. Enumeration data were expressed by rate, using Chi-square for comparisons. The univariate correlations between CEACAM1 and other parameters were performed both by Pearson’s and Spearman’s correlation analysis with adjustment of maternal age and gestational age. A two-tailed *P* < 0.05 was considered statistically significant. We calculated the sample size according to a GDM prevalence rate of approximately 8.1% in China [14]. Based on a previous study, the SD of CEACAM1 in healthy persons was 109.7 pg/ml [15]. In our study, 140 subjects including 70 GDM and 70 normal glucose tolerance pregnant subjects were enrolled. Using the current sample, to detect a 55 pg/ml difference with a significance level of 0.05 in GDM and NGT group, the power was 82.3% (α = 0.05), therefore, the sample size was considered to be adequate.

## Results

### Clinical and biochemical characteristics of the studied groups

As shown in Table 1, compared with NGT group, the GDM group had higher maternal age, FPG, 1hPG, 2hPG, FINS, 1hPINS, 2hPINS, HOMA-IR, AUCG, AUCI, neonatal weight and the percentages of positive family history of diabetes(all *P* < 0.05), while ISOGTT, △I60/△G60 of GDM group were significantly lower than the NGT group (all P < 0.05). Other parameters including blood pressure, pre-pregnancy BMI, pregnancy BMI, parity times, abdominal perimeter and HOMA-β were similar among the two groups (all *P* > 0.05). The circulating CEACAM1 levels in GDM group tended to be higher compared to NGT group, but the difference had no statistical significance (*P* > 0.05) (Table 1).

**Table 1:** Clinical characteristics and circulating CEACAM1 levels of the groups studied

Variable	GDM (*n* = 70)	NGT (*n* = 70)	*P* value
Age, years	28.54 ± 4.28	26.87 ± 3.79	0.016
Gestational age, weeks	24.66 ± 1.10	24.81 ± 1.12	0.404
Parity, times	1 (1–6)	2 (1–4)	0.058
BMI-1, kg/m2	21.54 ± 3.13	21.30 ± 3.00	0.605
BMI-2, kg/m2	24.35 ± 3.07	24.03 ± 3.21	0.673
Abdominal perimeter, cm	91.73 ± 6.17	90.20 ± 7.44	0.207
SBP, mm/Hg	113.40 ± 8.59	110.53 ± 8.54	0.061
DBP, mm/Hg	72.07 ± 6.32	70.79 ± 4.98	0.214
HbA1c, %	5.12 ± 0.76	4.96 ± 0.37	0.128
FPG, mmol/l	4.81 ± 0.68	4.36 ± 0.30	< 0.001
1hPG, mmol/l	9.60 ± 1.57	6.94 ± 1.34	< 0.001
2hPG, mmol/l	8.64 ± 1.55	6.26 ± 0.89	< 0.001
FINS, mU/l	4.88 (3.44–6.93)	3.22 (2.33–4.89)	0.002
1hPINS,mU/l	35.82 (20.83–46.77)	21.67 (14.36–31.90)	0.002
2hPINS,mU/l	32.30 (20.81–57.91)	23.30 (13.09–32.47)	< 0.001
HOMA-IR	1.03 (0.66–1.57)	0.65 (0.45–0.99)	0.004
HOMA-β	81.79 (60.05–118.38)	86.91 (54.10–131.61)	0.524
ISOGTT	151.00 (106.78–219.80)	278.89 (190.63–374.30)	< 0.001
AUCG, mmol/l	16.326 ± 2.254	12.251 ± 1.622	< 0.001
AUCI, mU/l	55.072 (39.09–75.00)	33.733 (23.51–54.53)	< 0.001
ΔI60/ΔG60	6.00 (3.75–8.01)	9.21 (4.89–14.56)	0.001
Neonatal weight, g	3477.78 ± 356.43	3333.13 ± 340.42	0.032
Family history of DM, %	10%	1.43%	< 0.001
CEACAM1, pg/ml	1889.82 ± 616.14	1758.92 ± 433.15	0.148

Note: Data are means ± standard deviation (SD) or medians (inter-quartile range)

Abbreviations: *BMI-1* body mass index before pregnancy, *BMI-2* body mass index in pregnancy, *DBP* diastolic blood pressure, *SBP* systolic blood pressure, *FPG* fasting plasma glucose, *1hPG* 1-hour post-meal plasma glucose, *2hPG* 2-h post-meal plasma glucose, *FINS* fasting insulin level, *1hPINS* 1-h post-meal plasma insulin level, *2hPINS* 2-h post-meal plasma insulin level, *AUCG* area under curve of glucose, *AUCI* area under curve of insulin

### Correlations of circulating CEACAM1 levels with anthropometric and biochemical parameters in GDM group

As shown in Table 2, in GDM group, univariate correlation analysis after adjustment of maternal age and gestational age showed that circulating CEACAM1 levels were positively correlated with ISOGTT (*R* = 0.39, *P* = 0.001), while negatively correlated with 1hPINS (*R* = -0.32, *P* = 0.008), 2hPINS (*R* = -0.33, *P* = 0.006), AUCI (*R* = -0.36, *P* = 0.002), no significant correlations were observed between CEACAM1 and the other parameters (*p* > 0.05).

**Table 2:** Correlations of CEACAM1 with anthropometric and biochemical parameters in GDM group

Variable	R	*P* value
SBP	−0.03	0.816
DBP	0.09	0.460
BMI1	0.06	0.649
BMI2	0.04	0.724
FPG	0.03	0.799
1hPG	−0.16	0.186
2hPG	−0.15	0.203
HbA1c	0.15	0.216
FINS	−0.03	0.835
1hPINS	−0.32	0.008
2hPINS	−0.33	0.006
HOMA-IR	−0.10	0.895
HOMA-β	−0.19	0.120
ISOGTT	0.39	0.001
AUCI	−0.38	0.002
AUCG	−0.16	0.186

Adjusted for age and gestational age

Note: Data are means ± standard deviation (SD) or medians (inter-quartile range)

Abbreviations: *BMI-1* body mass index before pregnancy, *BMI-2* body mass index in pregnancy, *DBP* diastolic blood pressure, *SBP* systolic blood pressure, *FPG* fasting plasma glucose, *1hPG* 1-h post-meal plasma glucose, *2hPG* 2-h post-meal plasma glucose, *FINS* fasting insulin level, *1hPINS* 1-h post-meal plasma insulin level, *2hPINS* 2-h post-meal plasma insulin level, *AUCG* area under curve of glucose, *AUCI* area under curve of insulin

## Discussion

In our study, we first determined circulating levels of CEACAM1 in GDM women. The important finding of the current study was that circulating CEACAM1 levels were associated with serum insulin levels and ISOGTT in the GDM group. Although circulating CEACAM1 levels were higher in the patients with GDM than that in the NGT group, however, we failed to observe statistically significant difference between two groups.

CEACAM1 protein was implicated in cell proliferation, apoptosis, angiogenesis and immune-regulation during tumor development [16]. In the past years, it had been studied as a tumor marker of different types of cancer. On the other hand, earlier studies indicated that CEACAM1 was also related to some metabolic diseases [17–19]. Previous articles reported that the hepatic CEACAM1 expression was markedly decreased in the severely obese subjects, high grade fatty livers and NASH, but not different between diabetic and non-diabetic persons [20]. Consistent with this study, some researchers also indicated that CEACAM1 mRNA levels were declined significantly in the liver of obese humans and obese rats, as expected, the obese rats then displayed hyperinsulinemia, elevated body weight, fasting plasma free fatty acid, and plasma and hepatic total triglycerides levels [21, 22]. These studies suggested that the decreased CEACAM1 levels might be an early event in obese subjects occurring at the time of insulin resistance before overt diabetes.

In our study, women with GDM showed higher FPG, FINS and HOMA-IR than the NGT group. This result demonstrated that GDM women were in a hyper-insulin state which was complicated with impaired glucose tolerance. As reported, progressive IR was thought to be the major factor in the pathogenesis of GDM, women with GDM had exaggerated IR compared to healthy pregnant women [23, 24]. While the exact mechanisms of IR were complicated and unclear. CEACAM1 regulated hepatic insulin clearance to maintain normoinsulinemia and insulin sensitivity [25]. So far, there were limited studies that had determined the CEACAM1 levels in pregnancy, and the relationships between GDM and CEACAM1 remained unclear. In regard to this, we conducted correlation analysis between CEACAM1 and metabolic parameters in GDM subjects. In this paper, we observed a strong positive association between circulating CEACAM1 and ISOGTT, while adversely negative correlation with AUCI, 1hPINS and 2hPINS when adjusting for age and gestational age in GDM subjects. These findings indicated that CEACAM1 might be closely related to serum insulin levels as well as insulin sensitivity in GDM women. In accordance with our results, earlier studies on experimental animal models reported that mice with null deletion of CEACAM1 or with liver-specific inactivation of CEACAM1 subsequently developed chronic hyperinsulinemia, insulin resistance, hepatic steatosis and visceral obesity [26–28]. Thus, abnormalities of insulin clearance may induce IR and then the occurrence of various pathological conditions including T2DM and severe obesity. Since CEACAM1 is a cytokine with insulin-sensitizing effects, it is likely that CEACAM1 levels would compensatory increase in GDM women to improve severe IR condition. However, in contrast to our hypothesis, this study observed that CEACAM1 levels were similar in both GDM and NGT group. A recent study reported that circulating CEACAM1 levels were different in the three trimesters during pregnancy, and the lowest levels were observed in the second trimester [29]. Given the fact that our blood samples were collected in the second trimester, it may be an explanation for the result.

The current study included some limitations. Firstly, we didn’t measure the blood lipid profile and analyze the possible associations between CEACAM1 and lipid metabolism. Secondly, we failed to investigate the longitudinal changes of circulating CEACAM1 levels during pregnancy and after delivery.

## Conclusions

Taken together, the results of this study showed a lack of significant difference in terms of CEACAM1 levels between GDM subjects and normal pregnant women. However, we found a significant positively correlation between CEACAM1 and insulin sensitivity after adjustment of age and gestational age in GDM group. Further studies with larger populations are needed to elucidate the role of CEACAM1 in the pathogenesis of GDM.

## Data Availability

The datasets used and/or analyzed during the current study are available from the corresponding author on reasonable request.

## Abbreviations

CEACAM1: Carcinoembryonic antigen-related cell adhesion molecule 1
GDM: Gestational diabetes mellitus
OGTT: Oral glucose tolerance test
BMI: Body mass index
DBP: Diastolic blood pressure
SBP: Systolic blood pressure
FPG: Fasting plasma glucose
FINS: Fasting insulin level
AUCG: Area under curve of glucose
AUCI: Area under curve of insulin
